# Environmental risk mapping of canine leishmaniasis in France

**DOI:** 10.1186/1756-3305-3-31

**Published:** 2010-04-08

**Authors:** Lise Chamaillé, Annelise Tran, Anne Meunier, Gilles Bourdoiseau, Paul Ready, Jean-Pierre Dedet

**Affiliations:** 1 Université Montpellier 1, Laboratoire de Parasitologie, Centre National de Référence des Leishmania, CHU de Montpellier and UMR 2724 GEMI (IRD-CNRS-UM1), Montpellier, France; 2 CIRAD, UR AGIRs, Montpellier, France; 3 CIRAD, UMR TETIS, Montpellier, France; 4 Unité de Parasitologie, Ecole Nationale Vétérinaire de Lyon, Marcy L'Etoile, France; 5 Department of Entomology, Natural History Museum, London, UK

## Abstract

**Background:**

Canine leishmaniasis (CanL) is a zoonotic disease caused by *Leishmania infantum*, a Trypanosomatid protozoan transmitted by phlebotomine sandflies. Leishmaniasis is endemic in southern France, but the influences of environmental and climatic factors on its maintenance and emergence remain poorly understood. From a retrospective database, including all the studies reporting prevalence or incidence of CanL in France between 1965 and 2007, we performed a spatial analysis in order to i) map the reported cases in France, and ii) produce an environment-based map of the areas at risk for CanL. We performed a Principal Component Analysis (PCA) followed by a Hierarchical Ascendant Classification (HAC) to assess if the locations of CanL could be grouped according to environmental variables related to climate, forest cover, and human and dog densities. For each group, the potential distribution of CanL in France was mapped using a species niche modelling approach (Maxent model).

**Results:**

Results revealed the existence of two spatial groups of CanL cases. The first group is located in the Cévennes region (southern Massif Central), at altitudes of 200-1000 m above sea level, characterized by relatively low winter temperatures (1.9°C average), 1042 mm average annual rainfall and much forest cover. The second group is located on the Mediterranean coastal plain, characterized by higher temperatures, lower rainfall and less forest cover. These two groups may correspond to the environments favoured by the two sandfly vectors in France, *Phlebotomus ariasi *and *Phlebotomus perniciosus *respectively. Our niche modelling of these two eco-epidemiological patterns was based on environmental variables and led to the first risk map for CanL in France.

**Conclusion:**

Results show how an ecological approach can help to improve our understanding of the spatial distribution of CanL in France.

## Background

Canine leishmaniasis (CanL) is a disease caused by *Leishmania infantum*, a Trypanosomatid protozoan transmitted by phlebotomine sandflies. This parasite also causes the human disease (zoonotic visceral leishmaniasis) throughout its worldwide range, including the Mediterranean Basin. The domestic dog is the main reservoir host, and this explains the socio-economic interest of the zoonosis [1]. CanL threatens a large number of dogs in endemic areas, and it is difficult to control as no efficient vaccine exists and the chemotherapeutic agents have a limited efficacy and a high cost [2]. Although CanL is endemic in southern France, it is not a notifiable disease nationally, which results in an absence of clear knowledge of its incidence and emergence. Up to now, the prevalence of CanL in France has been evaluated either directly through canine serological surveys [3,4], or indirectly through surveys by questionnaires to practising veterinarians [5]. Based on temporal surveys, CanL prevalence seems to have increased over the last decade [5,6]. For example, between 1988 and 2004, there was a doubling in the numbers of « départments » (the French administrative unit equivalent to a county) in which vets diagnosed more than 50 cases per year [5].

Nevertheless, it is difficult to distinguish between new cases resulting from local transmission by sandflies and those arising from dogs taken on holiday in the Mediterranean region [1]. Epidemiological surveillance and risk mapping of the disease require additional information and, since 2004, the EDEN EU FP6 project (Emerging Diseases in a changing European eNvironment: http://www.eden-fp6project.net) has been identifying and evaluating environmental conditions that can influence the spatial and temporal distribution of CanL and other vector-borne diseases. A retrospective CanL database was prepared by teams in many endemic European countries (France, Greece, Italy, Portugal and Spain), in order to carry out risk mapping using Geographic Information Systems (GIS). EDEN's risk map for CanL in Europe is based on a statistical approach using logistic regression, but here we present an ecological approach to modelling used only for France.

Two sandfly species are vectors of CanL in France, *Phlebotomus perniciosus *and *P. ariasi *[4,7]. However, each species has specific environmental associations [7]: *P. perniciosus *is present throughout Mediterranean France at altitudes less than 600 m above sea level (a.s.l.), while *P. ariasi *preferentially occurs in mixed oak forests (holm and downy oaks) 200-1400 m a.s.l. and it is less abundant on the Mediterranean littoral plain. This knowledge helped inform our choice of environmental variables for modelling.

## Methods

### Retrospective canine leishmaniasis database

The retrospective canine leishmaniasis database was specifically created within the EDEN project (Davies CR, Cox J and Ready PD, unpublished). The criteria for inclusion included any case report or study reporting prevalence or incidence of canine leishmaniasis in France between 1965 and 2007. The cases included were confirmed by parasitological, serological or molecular techniques. Imported cases were excluded from the database.

All data were entered into a single spreadsheet file. The data entered included the source of information, the type of survey or case reporting, the method of diagnosis used, information about the dog(s) concerned, and the location of the case(s) or survey(s), with geographical coordinates of the locality obtained using "Google Earth".

Mapping used GIS software (ESRI ArcGIS™) to observe distribution patterns and to facilitate statistical analyses.

### Environmental variables

The geographical distribution of CanL is related to environmental conditions that can influence the distribution and density of both the sandfly vector and the mammalian reservoir host [8]. The distribution of sandflies in France is strongly influenced by favoured Mediterranean vegetation zones [7] and climatic factors, e.g. seasonal temperatures [9]. Based on this knowledge, the following environmental variables were chosen as explanatory variables for CanL distribution: summer and winter precipitations, summer and winter temperatures, land use (in particular the type of forest) and altitude levels. Human and canine densities were also selected, although it should be noted that the latter was calculated using a different estimate of the former (Table 1).

**Table 1:** Environmental information used to characterize the CanL locations in southern France

Information	Variable (unit)	Data source (spatial resolution, date)
Altitude	Altitude (m)	Institut Géographique National (IGN) BDALTI database (250 m)
Temperature	Winter minimum temperature (°C): average of the normal minimum temperatures of January, February and March Summer maximum temperature (°C): average of the normal maximal temperatures of July, August and September Annual mean temperature (°C): annual average of the normal temperature	Météo France (Interpolation from values of normal of temperatures and precipitation of Météo France stations between 1971 and 2000. The method of interpolation is the method of the exponential ordinary kriging for the continent; the method is the inverse distance weighted for Corsica. They show the most suitable results compared with a set of map from Météo France)
	
Precipitation	Summer rainfall (mm): sum of rainfall of July, August and September Winter rainfall (mm): sum of rainfall of January, February and March Annual total rainfall (mm)	

Forest	Presence of three types of forest (Broadleaf forest, coniferous forest, mixed forest)	CORINE Land Cover (100 m, 2006)

Human density	Density per locality (number of residents divided by the surface of the locality)	IGN and Institut national de la statistique et des études économiques (INSEE) (locality, 2006)

Canine density	Density per locality (estimated number of dogs divided by the surface of the locality)	EDEN project http://edendatasite.com (0.008333°, 2005)

**Figure 1:**
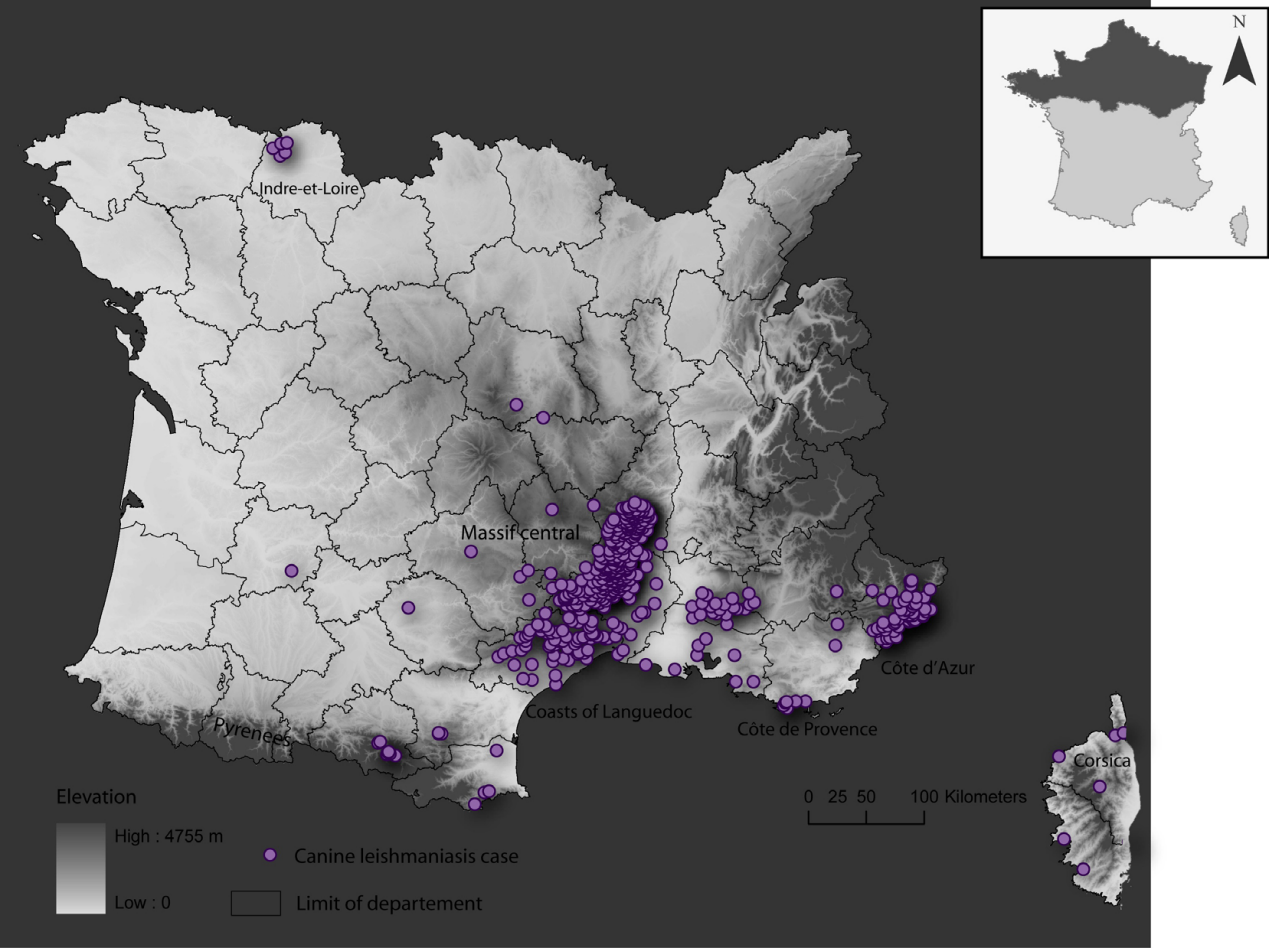
**Location in France of CanL cases for the 1965-2007 period**.

All variables were transformed, in order to be integrated into a GIS with the same projection (Lambert conformal conic projection) and the same geographical area (or mask) corresponding to the southern part of France, the grey area in Figure 1.

### Statistical analysis

In order to take into account the error of localization of the cases and to compare equivalent spatial units, we used a regular grid with 5 × 5 km cells for the analysis. This surface is equal to the average surface of the municipalities. A cell was considered to be endemic for CanL if it contained at least one locality with at least one CanL case.

A Principal Component Analysis (PCA) was carried out to generate an integrative description of the different characteristics of the cells, namely the following variables (Table 1):

- average altitude

- average annual temperature, average winter minimum temperature and average summer maximum temperature

- average annual, winter and summer rainfall

- percentages of surface covered by broadleaf forest, coniferous forest and mixed forest

- percentage of surface covered by forest (total forest)

- average human density

- average dog density

The PCA results in synthetic variables - Principal Components (PC) - which are a linear combination of the initial variables. By construction, there is no correlation between the resulting PCs, although two or more individual variables might be co-varying within a PC.

A Hierarchical Ascendant Classification (HAC) was performed on the PCs, allowing the cells with similar environmental characteristics to be grouped together. This classification method successively grouped together the cells, in order to obtain the most homogeneous and the most distinctive classes (groups) according to similarity and aggregation criteria [10]. The criterion of similarity was the Spearman coefficient and the criterion of aggregation was the average link.

### Ecological niche modelling

We used an ecological niche modelling approach to map the areas more suitable for the presence of the CanL in France. Various models of presence-only data are available to define the borders of potential ecological niches (see for example [11-15]). We chose a general-purpose machine learning method, the Maxent model, which has been recently demonstrated to offer better performance compared to other presence-only models [14,16]. Maxent is a method based on the maximal entropy principle. The model estimates the probability distribution (the probability of a case being present in each cell) that respects a set of constraints based on the values of the environmental variables observed for the occurrence data. Among all probability distributions that satisfy the set of constraints, the one with the maximum entropy is chosen. Unlike other species' modelling approaches, Maxent does not rely on any assumption of independence of the environmental variables, which is frequently not met for environmental data sets, and can incorporate interactions between different variables [16,17].

For each group identified by the HAC, a univariate correlation analysis was performed to select the environmental variables to be used as input of the Maxent model. The initial data set with all locations of reported CanL cases (presence-only data) was transformed into a relative density map (quantitative data), using a quadratic Kernel function [18]. The radius for the Kernel density estimates (0.1435°) was chosen following the method of Berman and Diggle [19]. The correlations between the case density and the different environmental variables were tested using the Pearson r correlation coefficient. Significant variables in this preliminary univariate screening analysis at a 0.1 p-value were then used in the Maxent procedure.

## Results

### Retrospective canine leishmaniasis database

The retrospective CanL database was produced between 2006 and 2008. It contains 718 entries, corresponding to 45 publications or sources and 425 locations.

The map of the locations corresponding to the presence of CanL since 1965 highlighted a spatial heterogeneity in the disease distribution (Figure 1). There were three clusters in southern France: i. on the foothills of the Cévennes mountains and other southern ranges of the Massif Central facing the Mediterranean; ii. on the southwest foothills of the Maritime Alps; and iii. on the hilly Côte d'Azur near the Italian border. Fewer cases were observed on the littoral plain of the Mediterranean, and cases were sparse in the south-west region, within the Massif Central, in Indre-et-Loire department and in Corsica.

### Statistical analysis

**Figure 2:**
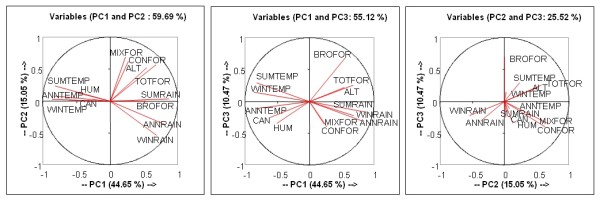
**Results of the Principal Component Analysis: composition of the Principal Components (PC1, PC2 and PC3) and projection of the initial variables on the first principal component analysis plans**. HUM: human density; CAN: canine density; ALT: altitude; BROFOR: broadleaf forest; CONFOR: Coniferous forest; MIXFOR: mixed forest; TOTFOR: total forest; SUMTEMP: summer temperature; WINTEMP: winter temperature; ANNTEMP: average annual temperature; SUMRAIN: summer rainfall; WINRAIN: winter rainfall; ANNRAIN: annual rainfall

The PCA was performed for 296 cells of 5 × 5 km, corresponding to the CanL case locations, coloured violet in our map of France (Figure 1). It resulted in 10 synthetic variables (PCs), with the first four factors summarizing about 80% of the observed variance. The first PC (PC1), which summarized more than 44% of the information, is a combination of temperature and precipitation variables. It can therefore be interpreted as a climatic factor. The second PC (PC2), summarizing 15% of the information, contains forest variables (coniferous, mixed forests and total forest), winter precipitation and altitude. The third PC (PC3, 10%) is mainly linked to broadleaf forest (Figure 2). The fourth PC (PC4, 8%) is linked to human and canine densities.

**Figure 3:**
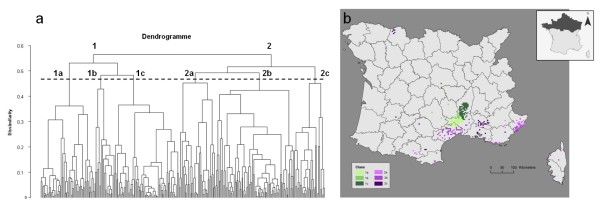
**Results of a Hierarchical Ascendant Classification of CanL cases according to environmental characteristics, 1965-2007, France**. a) dendrogram and b) map of the different classified groups.

The HAC of the individual coordinates of the PCA led to the successive grouping of the cells according to their environmental characteristics (Figure 3). It brought to light at least two important ecological profiles: cells located inland (Class 1) and those close to the coast (Class 2). Class 1 was positively associated with PC1. The cells of Class 1 corresponded to locations 200-1000 m a.s.l., which had the coldest winter temperatures (minimum winter temperatures between -0.6°C and 3.1°C, with an average of 1.9°C) and the highest precipitation (annual precipitation between 972 mm and 1254 mm, with an average of 1042 mm), and an important percentage of broadleaf forest. Class 2 included cells close to the mainland coast and in Corsica, with warmer summers (maximum summer temperatures between 23.4°C and 28°C, with an average of 26.1°C) and winters (minimum winter temperatures between 0.8°C and 6°C, with an average of 3.4°C) and less precipitation (annual precipitation between 362 mm and 1178 mm, with an average of 860 mm).

These two main classes may be divided into subclasses (Figure 3). Distinctions can be made between the cells of Class 1: sub-class 1a, positively associated with PC3 and PC4, contains more broadleaf forest as well as higher dog and human densities; sub-class 1b, positively associated with PC2, presents larger areas of coniferous and mixed forests; and sub-class 1c is negatively associated with PC2 and PC3 and thus contains less forest.

Class 2 can also be divided into three sub-classes: sub-class 2a, with a higher proportion of coniferous and mixed forests (correlated with PC2); sub-class 2b, with a lower proportion of forested areas (negatively correlated with PC2 and PC3); and sub-class 2c with a drier and warmer climatic profile combined with important areas of broadleaf forest.

### Ecological niche modelling

The Maxent model was run for the two main ecological profiles: Classes 1 and 2. According to the univariate analysis, seven significant environmental variables were selected as input for the Maxent model for Class 1: human and dog densities, average summer rainfall, average annual temperature, average winter minimum temperature, percentage of surface covered by coniferous forest, and altitude. For Class 2, five significant variables were selected: human density, average annual temperature, average winter minimum temperature, percentage of surface covered by broadleaf forest, and dog density (Table 2).

**Table 2:** Results of the univariate correlation analysis between CanL density and the environmental variables for two main ecological profiles

Class 1		
**Variable**	**Cor**	**p**

Human density	0.57	0.000

Summer rainfall	0.41	0.000

Canine density	0.32	0.000

Mean annual temperature	-0.21	0.019

Winter temperature	-0.21	0.021

Coniferous forest	0.19	0.044

Elevation	-0.17	0.062

Winter rainfall	-0.15	0.114

Mixed forest	0.14	0.122

Broadleaf forest	-0.13	0.155

Annual rainfall	0.10	0.290

Summer temperature	-0.06	0.523

Total forest	-0.04	0.664

**Class 2**		

**Variable**	**Cor**	**p**

Human density	0.34	0.000

Mean annual temperature	0.29	0.001

Winter temperature	0.28	0.002

Broadleaf forest	-0.19	0.029

Canine density	0.16	0.071

Total forest	-0.14	0.114

Annual rainfall	0.11	0.202

Winter rainfall	-0.09	0.310

Mixed forest	0.07	0.441

Summer temperature	0.06	0.507

Coniferous forest	0.046	0.624

Altitude	0.046	0.632

Summer rainfall	0.01	0.912

**Figure 4:**
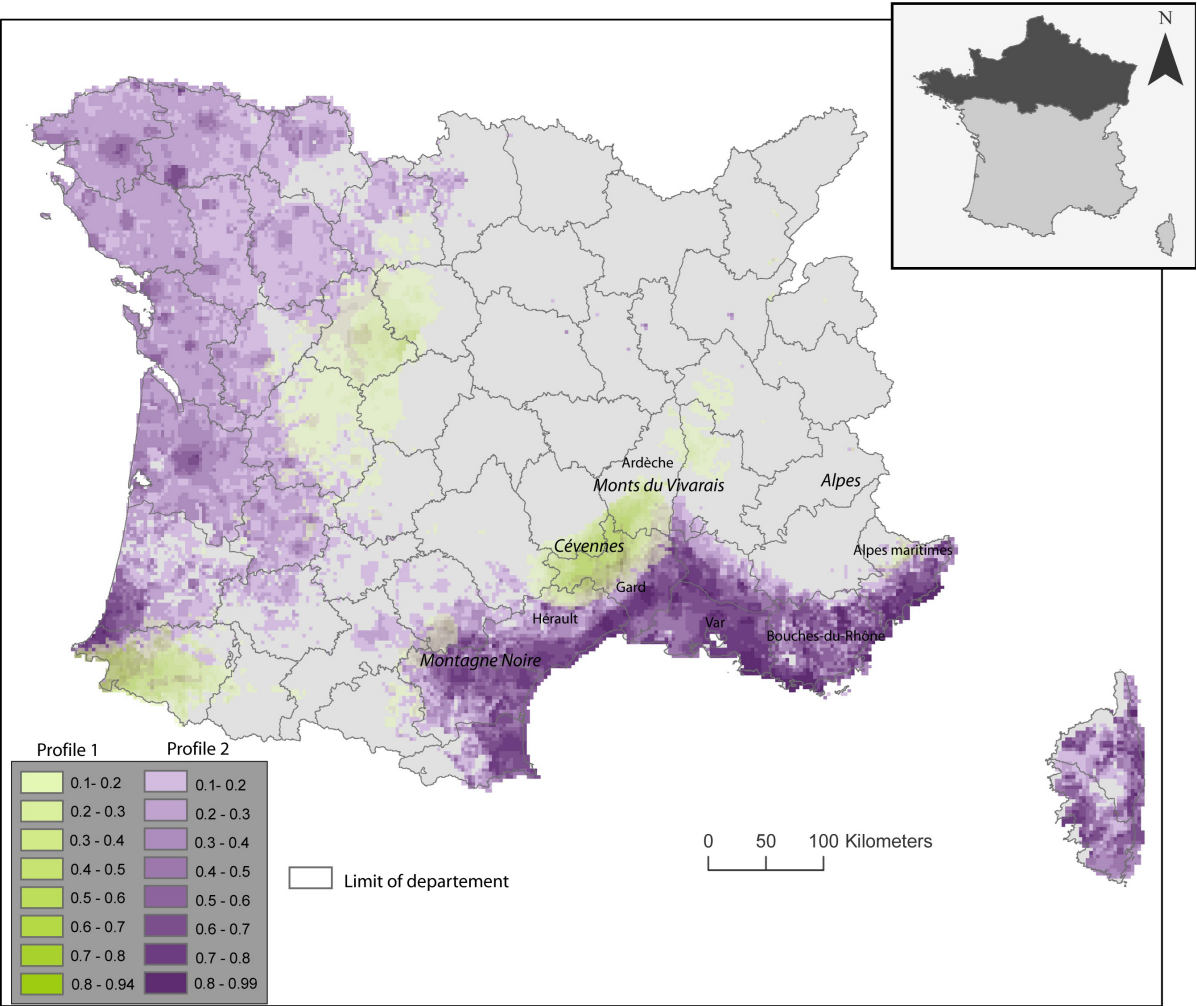
**Risk map of CanL in southern France**. The areas suitable for the transmission of CanL by the sandfly species *P. ariasi *and *P. perniciosus *are coloured in green and violet, respectively. The risk is expressed as a probability of occurrence with values ranging from 0 to 1.

The final risk map (Figure 4) was produced by superimposing the results of the Maxent model for Class 1 and 2. It showed an unequal distribution of the area suitable for the disease in the southern part of France: the most suitable areas extended along the southern slopes of the Cévennes from the Montagne Noire in the southwest to Monts du Vivarais in the northeast, and along the Mediterranean coast, particularly in the central and the eastern part of this littoral region. The main risk area for CanL in France included the Ardèche, Gard, Hérault, Bouches-du-Rhône, Var and Alpes-Maritimes départements. Several potentially suitable areas occurred on the western part of the Mediterranean coast and in the extreme southwest (Pays Basque).

Less suitable areas were the Alps, the Massif Central and the northern Rhône valley.

## Discussion

For the first time, a retrospective study of CanL in France has been carried out, based on cases reported between 1965 and 2007. The map of cases highlights a strong heterogeneity in the spatial distribution of the disease. Visually, the distribution of CanL in southern France is clustered, with higher case densities on the southern slopes of the Cévennes Mountains and two regions of the Maritime Alps (Figure 1). In addition to these Mediterranean records, this case map also shows a northern focus, corresponding to 13 cases detected by Houin *et al*. [20] in six different localities near Tours.

The case map is based on presence only, which does not takes into account the prevalence data obtained by some surveys. Some biases could not be avoided. Firstly, a single case report has the same value as a locality with high disease prevalence. Secondly, the clustering of presence spots might reflect the spatial distribution of the disease, and/or the sampling effort and strategy of the leishmaniasis teams from Montpellier, Lyon, Marseilles and Nice. Certainly, some areas were insufficiently reported, such as in the Pyrénées-Orientales département, where all the specific case localities were not noted in a publication giving an overall prevalence of 6.9% [21].

The statistical environmental analysis (PCA followed by a HAC) revealed the existence of two groups of leishmaniasis cases. The first group is located on the Cévennes slopes, characterized by relatively low average temperatures, high average rainfall and much forest cover. The second group is located on the Mediterranean coast, characterized by higher average temperatures, lower average rainfall and less forest cover (Figure 2). These two groups may correspond to the environments favoured by the two species of sandfly vectors in France, as previously shown in southern France[7] and Morocco [22,23]. Rispail et al. [23] identified, in Morocco, different associations between the distributions of the two vectors and Mediterranean bioclimatic zones, namely humid and sub-humid for *P. ariasi*, compared with sub-humid and semi-arid for *P. perniciosus*. Our environmental model also identified two distinctive profiles, with the two main classes matching the bioclimates associated with the two vector species: Class1 matches the bioclimates of *P. ariasi*, whereas Class 2 matches those of *P. perniciosus*.

According to the univariate correlation analysis, human and canine densities are, as we expected, significant variables for explaining the distribution of CanL in France. Their densities co-vary (Spearman r = 0.84), but both were retained to provide all relevant information about CanL distribution. This was possible because the Maxent procedure does not require independent variables. Average annual temperature and winter temperature also helped to define both environmental profiles, but in different ways: the number of CanL cases was negatively correlated with the temperature averages in the first profile (*P. ariasi)*, whereas it was positively correlated in the second profile (*P. perniciosus)*. Moreover, additional significant variables were selected for *P. ariasi*: the average summer rainfall, the proportion of coniferous forest and the elevation. On the other hand, the distribution of *P. perniciosus *was negatively correlated with the proportion of broadleaf forest. These differences are also consistent with the ecological niches of these two sandflies [7].

Our ecological niche modelling approach has produced the first risk map of CanL for France, highlighting the potential distribution of the disease. The new areas at risk are mostly located in western France, along the Atlantic coast, from the Pyrénées-Atlantique in the South to the Loire-Atlantique in the North. These areas correspond mainly to areas likely to be favoured by *P. perniciosus*, with only a few places having the ecological profile of *P. ariasi *(Haute-Vienne and Pyrénées-Atlantiques) (Figure 4). Some new areas at risk have ecological niches likely to be favourable for both species, but they are rare (Figure 4).

This risk map is consistent with the known distribution of *P. perniciosus*. Moreover, it should be noted that, in recent years, CanL cases have been reported in several locations outside the Mediterranean region, and always inside the Atlantic area where the risk map predicts emergence. These cases were reported around Limoges (Haute-Vienne) [24], near Cholet and Angers (Maine-et-Loire) (Bourdeau, 2009, personnal communication), and around Niort (Deux-Sèvres) (Kasbari, 2009, personal communication). In these places, a few imported CanL cases seem to be at the origin of local dog transmission, and horizontal dog transmission cannot be ruled out because cases were always grouped inside kennels. However, vectors were present as well.

Our risk map does not match well the range extensions of CanL mapped by Bourdeau [5]. The latter is based on vet questionnaires, and it shows a more limited range extension of clinical autochthonous cases of CanL to the north and west of the enzootic Mediterranean region. Our ecological niche model predicts the environmental suitability for CanL, separating this into two classes that probably reflect the niches of the two vectors, but the realized niche may be smaller than the fundamental niche predicted by the model [16].

The areas identified at risk for the disease may be used for entomological or veterinary surveillance. However, our results have to be treated with caution. Indeed, the risk model is based on a retrospective database concerning all reported cases of CanL. The assignment of the case locations (often the centre of the municipality) is likely to introduce some errors. For example, some cases recorded from the littoral plain of the Languedoc could be related to hunters' dogs, which could have contracted the disease in the Cévennes Mountains, where they are taken for hunting [3]. Subclass 2b could correspond to the leishmaniasis cases in this population of dogs (Figure 3).

## Conclusions

This paper shows how an ecological approach can help to improve our understanding of the spatial distribution of CanL in France. Our environmental risk map is the first to be produced and proved to be a useful tool for formulating hypotheses about CanL emergence. Further studies are needed to better understand the ecology of CanL in France. In particular, surveys to investigate the ecology of both sandfly vectors, *P. ariasi *and *P. perniciosus*, would help to interpret our risk maps. For example, studies of presence-absence of these sandflies in a smaller area could identify specific environmental variables (including land cover) that might be important predictors at local scales.

## Competing interests

The authors declare that they have no competing interests.

## Authors' contributions

LC and AT performed the statistical analyses of the data and the ecological niche modelling; AM and GB helped complete the retrospective database; PR was the coordinator of the leishmaniasis component of the EDEN project; J-PD directed the French team and helped develop the retrospective database. The manuscript was written by AT, LC, J-PD and PR.
